# The TPR Domain in the Host Cyp40-like Cyclophilin Binds to the Viral Replication Protein and Inhibits the Assembly of the Tombusviral Replicase

**DOI:** 10.1371/journal.ppat.1002491

**Published:** 2012-02-09

**Authors:** Jing-Yi Lin, Venugopal Mendu, Judit Pogany, Jun Qin, Peter D. Nagy

**Affiliations:** Department of Plant Pathology, University of Kentucky, Lexington, Kentucky, United States of America; Pasteur Institute, France

## Abstract

Replication of plus-stranded RNA viruses is greatly affected by numerous host-coded proteins acting either as susceptibility or resistance factors. Previous genome-wide screens and global proteomics approaches with *Tomato bushy stunt* tombusvirus (TBSV) in a yeast model host revealed the involvement of cyclophilins, which are a large family of host prolyl isomerases, in TBSV replication. In this paper, we identified those members of the large cyclophilin family that interacted with the viral replication proteins and inhibited TBSV replication. Further characterization of the most effective cyclophilin, the Cyp40-like Cpr7p, revealed that it strongly inhibits many steps during TBSV replication in a cell-free replication assay. These steps include viral RNA recruitment inhibited via binding of Cpr7p to the RNA-binding region of the viral replication protein; the assembly of the viral replicase complex and viral RNA synthesis. Since the TPR (tetratricopeptide repeats) domain, but not the catalytic domain of Cpr7p is needed for the inhibitory effect on TBSV replication, it seems that the chaperone activity of Cpr7p provides the negative regulatory function. We also show that three Cyp40-like proteins from plants can inhibit TBSV replication *in vitro* and Cpr7p is also effective against Nodamura virus, an insect pathogen. Overall, the current work revealed a role for Cyp40-like proteins and their TPR domains as regulators of RNA virus replication.

## Author Summary

Replication of plus-stranded RNA viruses, which are important pathogens of humans, animals and plants, can be inhibited by host-coded proteins. In this paper, the authors show that the Cyp40-like Cpr7p prolyl isomerase of yeast can effectively inhibit tombusvirus replication. This inhibition is due to binding of the TPR (tetratricopeptide repeats) domain of Cpr7p to the RNA-binding region of the tombusvirus replication proteins that leads to inhibition of RNA binding by the viral replication proteins, interference with the assembly of the viral replicase and blocking viral RNA synthesis. Cpr7p is also effective against the distantly-related alfanodaviruses of insects. Overall, this work reveals a role for a Cyp40-like protein as a regulator of RNA virus replication. This function of Cyp40 during RNA virus infection seems to be conserved between yeast and plants.

## Introduction

Replication of plus-stranded (+)RNA viruses takes place in membrane-bound viral replicase complexes (VRCs) in the cytoplasm of infected cells. (+)RNA viruses usurp a number of host-coded proteins to aid the replication process [1]–[8]. Many host proteins, however, have antiviral activities by inhibiting various steps of viral replication and infection. Accordingly, genome-wide screens to identify host factors affecting (+)RNA virus infections, such as *Tomato bushy stunt virus* (TBSV), West Nile virus, *Brome mosaic virus* (BMV), Hepatitis C virus (HCV), Dengue virus and Droshophila virus C in yeast or animal cells led to the identification of stimulatory as well as inhibitory host proteins [9]–[17]. The functions of the majority of the identified host proteins in (+)RNA virus replication have not been fully revealed.

TBSV is a small (+)RNA virus that has recently emerged as a model virus to study virus replication, recombination, and virus - host interactions due to the development of yeast (*Saccharomyces cerevisiae*) as a model host [18]–[21]. Genome-wide screens of yeast genes and global proteomics approaches have led to the identification of over 300 host genes/proteins that affected either TBSV replication or recombination [9], [11], [22], [23]. Also, proteomics analysis of the highly purified tombusvirus replicase complex revealed the presence of the two viral replication proteins (i.e., p33 and p92^pol^) and 6–10 host proteins in VRC [24], [25], [26]. These host proteins have been shown to bind to the viral RNA and the viral replication proteins [1], [25], [27]. For example, heat shock protein 70 (Hsp70), eukaryotic elongation factor 1A (eEF1A) and the ESCRT (endosomal sorting complexes required for transport) family of host proteins are required for the assembly of VRC, while glyceraldehyde-3-phosphate dehydrogenase (GAPDH) and eEF1A have been shown to affect viral RNA synthesis [27]–[31]. The auxiliary p33 replication protein has been shown to recruit the TBSV (+)RNA to the site of replication, which is the cytosolic surface of peroxisomal membranes [32]–[34]. The RdRp protein p92^pol^ binds to the essential p33 replication protein that is required for assembling the functional VRC [20], [34]–[36]. In contrast, the roles of identified host proteins with inhibitory functions in TBSV replication are less characterized. Nucleolin, an RNA binding protein, has been shown to interfere with the recruitment of the viral RNA into replication [37], while Rsp5p, a Nedd4 family of E3 ubiquitin ligase, regulates the degradation of p92^pol^ in yeast cells and the activity of VRC *in vitro* [38]. In addition, Cpr1p cyclophilin and Ess1p parvulin also decrease TBSV RNA accumulation in yeast, but the mechanism is not yet unraveled [39].

Cyclophilin family of proteins are ubiquitous, highly conserved proteins with prolyl isomerase (PPIase) activity. Cyclophilins and the structurally unrelated FKB proteins (FK506-binding proteins) and parvulins constitute a family of 13 prolyl isomerases in yeast. These proteins catalyze *cis*-*trans* isomerization of the peptidyl-prolyl bonds that could alter the structure, function or localization of the client proteins [40]–[41]. The isomerization of the peptidyl-prolyl bonds are frequently required for protein refolding after trafficking through cellular membranes [41]. The best-known member of prolyl isomerases in eukaryotes is cyclophilin A (CypA in mammals and Cpr1p in yeast). The most important functions of cyclophilins in cells are in protein folding, assembly of multidomain proteins, muscle differentiation, detoxification of reactive oxygen species, and immune response. Cyclophilins have been implicated in various diseases, such as cancer, atherosclerosis, diabetes and neurodegenerative diseases [40], [42], [43]. Because of their roles in immunosuppression, cyclophilins and FKBs are also called immunophilins.

To determine the mechanism of cyclophilin-based inhibition of tombusvirus replication, in this work, we first identified those cyclophilins, which interacted with the TBSV p33 replication protein, followed by testing the effect of cyclophilins in a cell-free tombusvirus replication assay. Further analysis of Cpr7p, which, among cyclophilins, is the strongest inhibitor of TBSV replication in yeast and *in vitro*, revealed that it binds to the RNA-binding domain of p33. This binding by Cpr7p, via its TPR (tetratricopeptide repeats) domain, leads to inhibition of p33/p92-based recruitment of the TBSV RNA for replication and decreases the efficiency of the VRC assembly. Thus, Cpr7p is a negative regulator of TBSV replication. We also show that Cpr7p can inhibit the replication of the insect alfanodaviruses in yeast model host, suggesting that Cyp40-like cyclophilins might have broad antiviral activities during RNA virus infections.

## Results

### Potent inhibition of TBSV replication in a cell-free replication assay by the p33-interacting members of the cyclophilin family

**Figure 1:**
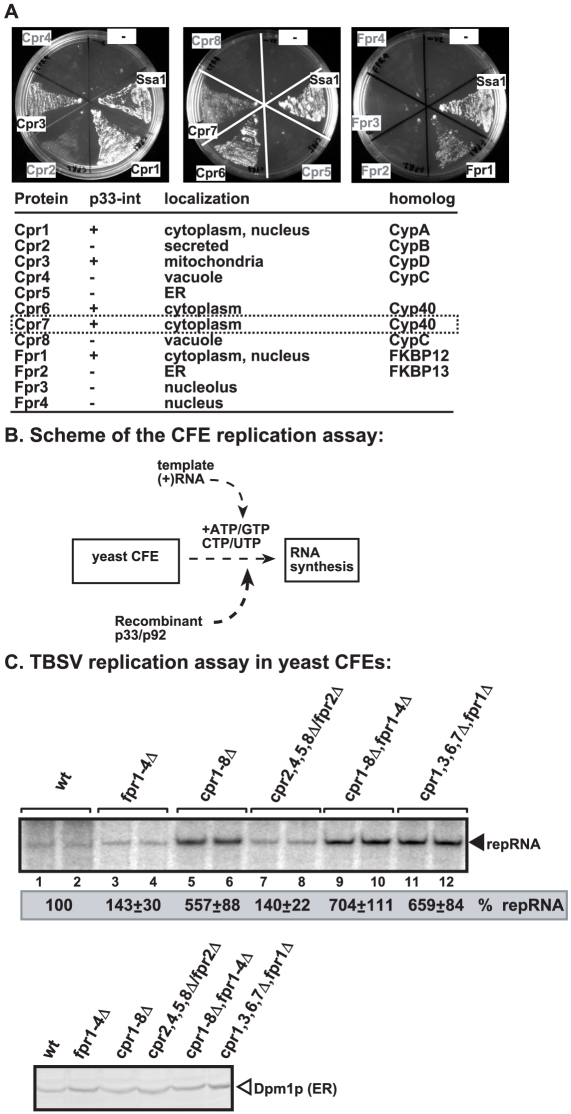
Identification of the members of the cyclophilin (immunophilin) family that interact with p33 and inhibit TBSV replication *in vitro*. (A) Split ubiquitin MYTH assay was used to test binding between p33 and the shown full-length yeast proteins. The bait p33 was co-expressed with the prey proteins in yeast. SSA1 (HSP70 chaperone), and the empty prey vector (NubG) were used as positive and negative controls, respectively. The interaction of cyclophilins with p33, their localization in cells and human homologs are summarized below the image. (B) Scheme of the CFE-based TBSV replication assay. Purified recombinant p33 and p92^pol^ replication proteins of TBSV and *in vitro* transcribed TBSV DI-72 (+)repRNA were added to the whole cell extract prepared from various yeast strains with deletion of selected members of the immunophilin genes as shown in panel C. (C) Top panel: Denaturing PAGE analysis of the ^32^P-labeled TBSV repRNA products obtained in the TBSV replication assay based on various CFEs as shown. Each experiment was repeated three times. Bottom panel: Western blot analysis of various CFEs with anti-Dpm1 antibody, an ER-resident protein, to show similar protein content in CFEs.

A previous screen based on MYTH (membrane yeast two-hybrid) split-ubiquitin assay and a yeast cDNA library has identified Cpr1 cyclophilin (CypA-like) as a strong interactor with p33 replication protein [39]. Since cyclophilins, together with the structurally unrelated FKB proteins and parvulins, constitute a family of 13 prolyl isomerases in yeast, we wanted to know if other members of PPIases could also interact with the TBSV replication proteins. The MYTH assay revealed that 6 members of the cyclophilin family, namely Cpr1p, Cpr3p, Cpr6p, Cpr7p, Fpr1p (Figure 1A) and Ess1p [39], interact with p33 replication protein.

To test if cyclophilins can directly affect the activity of the tombusvirus replicase, we prepared cell-free extracts (CFE) from yeast strains with deletion of multiple cyclophilin genes (Figure 1B). These yeast extracts contained comparable amount of total proteins (not shown) or an ER-resident marker protein [Dpm1p, [29]] (Figure 1C). The advantage of the CFE extract-based TBSV replication assay is that the CFE can be programmed with the TBSV (+)repRNA in the presence of purified recombinant p33 and p92^pol^ obtained from *E. coli* that leads to the *in vitro* assembly of the viral replicase, followed by a single cycle of complete TBSV replication, resulting in both (−)-stranded and (+)-stranded repRNA progeny [31], [35].

Using CFE prepared from a yeast strain missing all 8 *CPR* and 4 *FPR* genes (*ESS1* is an essential gene and thus it could not be deleted) resulted in ∼7-fold increase in TBSV repRNA accumulation *in vitro* (Figure 1C, lanes 9–10). This result firmly established that in general cyclophilins are strong inhibitors of TBSV replication. Using CFEs from yeast missing all 8 *CPR* genes also resulted in ∼5.5-fold increase in TBSV repRNA accumulation *in vitro* (Figure 1C, lanes 5–6). In contrast, CFE missing all 4 *FPR* genes supported TBSV replication only a little better than CFE from wt yeast (Figure 1C, lanes 3–4), suggesting that *FPRs* are not as important inhibitory proteins as *CPRs*. Also, CFE missing the five cyclophilins that interact with p33 (i.e., Cpr1p, Cpr3p, Cpr6p, Cpr7p, Fpr1p) supported TBSV replication ∼6.5-fold more efficiently than wt (Figure 1C, lanes 11–12), while deletion of those that do not interact (Cpr2p, Cpr4p, Cpr5p, Cpr8p, Fpr2p) resulted in close to wt level of TBSV replication (Figure 1C, lanes 7–8), suggesting that only the p33-interacting cyclophilins are important for inhibition of TBSV replication *in vitro*.

### The TPR domain of *CPR7* is a potent inhibitor of tombusvirus replication

**Figure 2:**
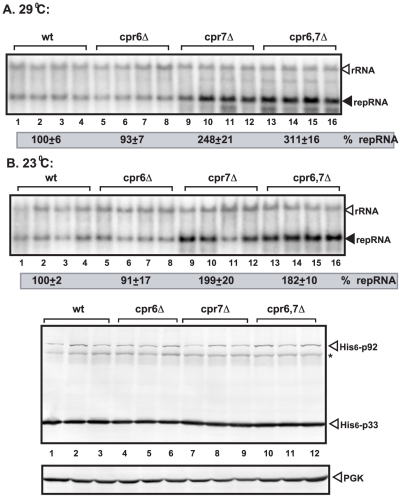
Increased TBSV repRNA accumulation in *cpr7*Δ yeast. (A) To launch TBSV repRNA replication, we expressed 6xHis-p33 and 6xHis-p92 from the copper-inducible *CUP1* promoter and DI-72(+) repRNA from the galactose-inducible *GAL1* promoter in the parental (BY4741) and in *cpr6Δ*, and *cpr7*Δ single deletion and *cpr6*Δ *cpr7*Δ double deletion yeast strains. The yeast cells were cultured for 40 hours at either 29°C (panel A) or 23°C (panel B) on 2% galactose SC minimal media. Northern blot analysis was used to detect DI-72(+) repRNA accumulation. The accumulation level of DI-72(+) repRNA was normalized based on 18S rRNA. Bottom panels: Western blot analysis of the accumulation level of 6xHis-tagged p33 and 6xHis-tagged p92 proteins using anti-His antibody. Asterisk marks the SDS-resistant p33 homodimer. The Western blot for PGK host protein at the bottom shows the loading control.

To identify which *CPRs* are the most potent inhibitors of TBSV replication, first we have tested TBSV repRNA replication in CFE when purified recombinant cyclophilins were separately added to the reaction. These CFE-based experiments revealed that recombinant Cpr3p and Cpr7p inhibited (Figure S1A, lanes 9–14), while Cpr1p (lanes 6–8) and Cpr6p (Figure S1C, lanes 6–8) did not inhibit TBSV repRNA accumulation *in vitro*. Additional testing of TBSV repRNA accumulation in single and double *CPR* deletion yeast strains revealed that *CPR7* is the strongest inhibitor (Figure 2 and not shown). Indeed, we observed that deletion of *CPR7*, which is a Cyp40-like cyclophilin that carries both the catalytic Cyp and TPR domains (involved in protein-protein interactions), in yeast resulted in the largest, ∼2–2.5-fold increase in TBSV repRNA accumulation (Figure 2A–B) among the single deletion strains (Figure 2 and not shown) [39]. Interestingly, deletion of *CPR6*, which is the second member of the Cyp40-like yeast cyclophilins, did not affect TBSV replication (Figure 2A–B), suggesting that, in spite of the similarities in protein sequence, *CPR7* and *CPR6* do not have overlapping function in inhibition of TBSV accumulation. This conclusion was further strengthened by the double-deletion yeast strain (*cpr6*Δ *cpr7*Δ), which supported TBSV accumulation at ∼2–3-fold higher level than that in the WT yeast, similar to the level seen in *cpr7*Δ (Figure 2A–B).

**Figure 3:**
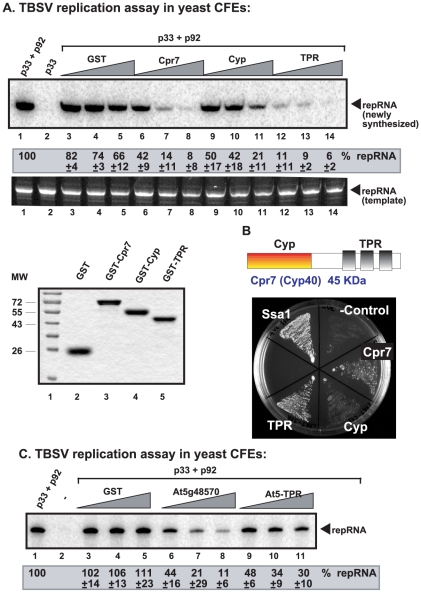
Cell-free TBSV replication assay supports an inhibitory role for Cpr7p and an *Arabidopsis* Cyp40 homolog. (A) Top panel: Denaturing PAGE analysis of the ^32^P-labeled TBSV repRNA products obtained in the CFE-based assay programmed with *in vitro* transcribed TBSV DI-72 (+)repRNA and purified recombinant p33 and p92^pol^ replication proteins of TBSV. Purified recombinant GST-tagged Cpr7p, the Cyp domain or the TPR domain (0.4, 0.8 and 1.6 µg), or GST were added to CFE prepared from BY4741 yeast strain. Each experiment was repeated three times. Middle panel: Ethidium-bromide stained PAGE gel from the top panel to show the sample loading and the lack of RNase activity in the CFE-based assay. Bottom panel: SDS-PAGE analysis of the purified recombinant proteins used in the above CFE-based assay. (B) Split ubiquitin assay was used to demonstrate binding between p33 and the TPR and the catalytic Cyp domains of Cpr7p. The domain structure and size of Cpr7p is shown on the top. (C) Denaturing PAGE analysis of the ^32^P-labeled TBSV repRNA products obtained in the CFE-based TBSV replication assay containing purified recombinant *Arabidopsis* Cyp40 homolog At5g48570 and its TPR domain (0.4, 0.8 and 1.6 µg).

To measure the direct effect of Cpr7p on TBSV RNA accumulation, we performed CFE-based TBSV replication assays with added purified recombinant Cpr7p. The inhibition of TBSV RNA replication by Cpr7p reached up to 92% *in vitro* (Figure 3A, lane 8). The purified TPR domain of Cpr7p was even more potent inhibitor of TBSV replication *in vitro* (Figure 3A, lanes 12–14) than the full-length protein, while the catalytic Cyp domain had a lesser effect (up to 80%, Figure 3A, lane 11).

The MYTH-based interaction of Cpr7p and its domains with p33 showed that the TPR domain is the strongest interactor, while the full-length protein was also a good interactor when compared with the Cyp-domain (Figure 3B). Thus, there seems to be correlation between the strength of interaction and extent of inhibition by Cpr7 and its derivatives on TBSV replication *in vitro*. Interestingly, this is not the case with Cpr6p, which strongly interacted with p33, but did not inhibit TBSV replication effectively (Figure S2A). Data shown below in the paper, however, can explain why *CPR6* deletion in yeast did not increase TBSV replication as much as deletion of *CPR7*.

### The Cyp40-like proteins and their TPR domains from *Arabidopsis* are inhibitors of tombusvirus replication

To determine if plant Cyp40-like proteins can also inhibit tombusvirus replication, we cloned, expressed and purified the TPR domains of three *Arabidopsis thaliana* Cyp-40-like proteins and two full-length proteins, followed by testing them in our CFE-based TBSV replication assay (Figure S3). We found that the TPR domains of all three *Arabidopsis* Cyp40 proteins inhibited TBSV replication. The most efficient inhibitor was the full-length At5g48570 Cyp40 protein by reducing TBSV RNA accumulation by ∼90% *in vitro* (Figure 3C, lane 8 versus lane 1).

The split-ubiquitin assay revealed that the TPR-domain of At5g48570 and AtTWD1 interacted strongly with both p33 and p92, while At2g15790 interaction was weak, but detectable (Figure S3B–C). Altogether, these data indicate that the plant Cyp40 proteins and their TPR domains interact with TBSV p33/p92 replication proteins and At5g48570 is as potent inhibitor of tombusvirus replication as the yeast Cpr7p.

### The mechanism of *CPR7*-based inhibition of tombusvirus replication

**Figure 4:**
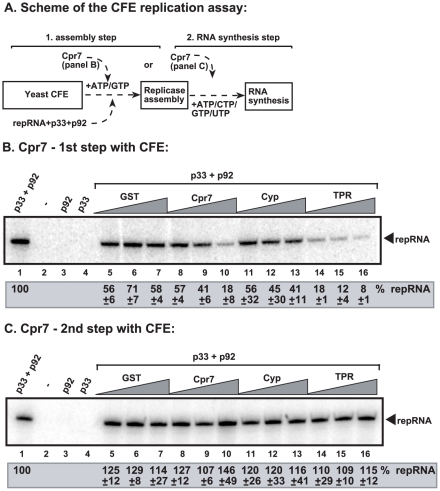
Step-wise CFE-based TBSV replication assay supports an inhibitory role for Cpr7p in the assembly of the TBSV VRC. (A) Scheme of the CFE-based TBSV replicase assembly and replication assays. Purified recombinant p33 and p92^pol^ replication proteins of TBSV and *in vitro* transcribed TBSV DI-72 (+)repRNA were added to CFE prepared from BY4741 yeast strain in step 1. The assay either contained or lacked the purified recombinant Cpr7p, its derivatives (0.4, 0.8 and 1.6 µg), or GST during step 1. Note that the assay was performed in the presence of ATP/GTP to facilitate TBSV VRC assembly, but prevent RNA synthesis in step 1. After step 1, centrifugation was used to collect the membrane fraction of the CFE, and after washing the membranes, step 2 was performed in the presence of ATP/CTP/GTP and ^32^P-UTP to allow TBSV RNA replication. In the samples presented on the right side of the panel, the recombinant Cpr7p or derivatives were added at the beginning of step 2. (B) Denaturing PAGE analysis of the ^32^P-labeled TBSV repRNA products obtained in the CFE-based assays when Cpr7p or derivatives were added at the 1st step. See further details in Figure 1. (C) Denaturing PAGE analysis of the ^32^P-labeled TBSV repRNA products obtained in the CFE-based assays when Cpr7p or derivatives were added at the 2nd step. Three repeats of each experiment were performed.

Since the recombinant Cpr7p was a potent inhibitor of TBSV replication *in vitro*, we used our CFE-based approach to further dissect the mechanism of Cpr7p-mediated inhibition. Briefly, a two-step replication assay based on yeast CFE was used to determine what steps of TBSV replication could be inhibited by Cpr7p. In this assay, the first step includes the assembly of the replicase complex on the endogenous membranes present in CFE in the presence of the viral (+)repRNA, the recombinant p33 and p92 replication proteins and ATP/GTP [31] (Figure 4A, step 1). Under these conditions, the viral replication proteins recruit the (+)repRNA to the membrane and the viral replicase becomes partially RNase and protease insensitive. However, the assembled replicase cannot initiate minus-strand synthesis yet, due to the absence of CTP/UTP [31]. Then, centrifugation and washing the membranes will remove all the proteins and molecules not bound to the membrane. This is then followed by addition of ATP/CTP/GTP/UTP (^32^P-labeled) to initiate RNA synthesis during the second step.

Addition of purified recombinant Cpr7p to the CFE during the first step inhibited TBSV replication up to ∼80% (Figure 4B, lane 10 versus 1), similar to the strong inhibitory effect of Cpr7p during standard CFE replication assay (Figure 3). This suggests that Cpr7p might inhibit the assembly of the replicase complex. Interestingly, the TPR domain was again a more potent inhibitor (Figure 4B, lanes 14–16) than the full-length Cpr7p, while the Cyp domain was a lesser inhibitor (Figure 4B, lanes 11–13). However, addition of Cpr7p or its truncated mutants exclusively during the second step of the CFE assay did not inhibit TBSV RNA replication (Figure 4C). The lack of inhibition by Cpr7p during the second step suggests that either Cpr7p cannot inhibit RNA synthesis by the pre-assembled replicase or Cpr7p cannot access, and thus cannot inhibit the membrane-bound replicase (possibly due to physical separation or constrainst by the membranous structure).

**Figure 5:**
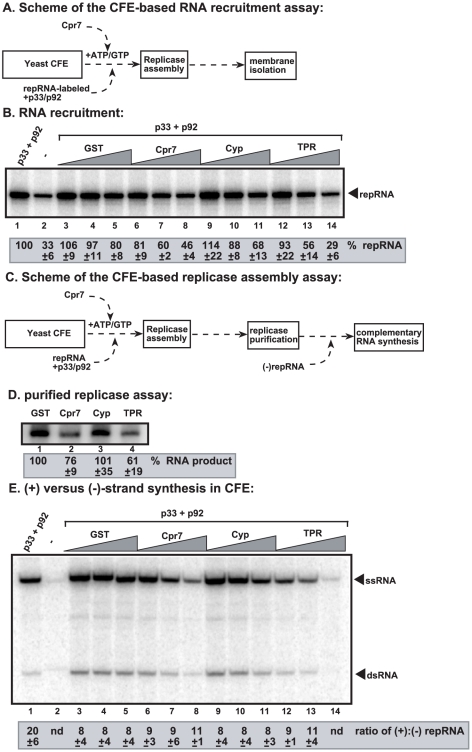
Cpr7p affects multiple steps during TBSV RNA replication *in vitro*. (A) Scheme of the viral RNA recruitment assay based on CFE. The ^32^P-labeled TBSV (+)repRNA template was added together with p33/p92 and Cpr7p or its derivatives to CFE prepared from BY4741 yeast. The membrane-association of ^32^P-labeled TBSV (+)repRNA template is measured using denaturing PAGE gels (Panel B). Note that ^32^P-labeled TBSV (+)repRNA template can inefficiently associate with the membrane even in the absence of p33/p92 (lane 2), likely due to nonspecific binding to an RNA-binding host protein in the membrane. (C) Scheme of the CFE-based TBSV replicase assembly assay. Note that the original template RNA is removed during replicase solubilization/purification. Therefore, an added (−)repRNA is tested for replicase activity for each replicase prep. (D) Complementary RNA synthesis that results in ^32^P-labeled TBSV (+)repRNA template is measured using denaturing PAGE gels. (E) Detection of single- and double-stranded RNA products produced in the CFE-based TBSV replication assay. The ratio of ssRNA and dsRNA in the samples are shown. Note that the dsRNA product represents the annealed ^32^P-labeled (−)RNA and the (+)RNA, while the ssRNA products represents the newly made ^32^P-labeled (+)RNA products.

To systemically dissect what steps in TBSV replication are inhibited by Cpr7p, first we performed viral (+)RNA recruitment assay (Figure 5A). The recruitment of the cytosolic TBSV (+)repRNA to the peroxisomal or ER membrane surfaces, where replication takes place, requires p33 and p92 replication proteins [29], [33], [44]. We found the purified recombinant Cpr7p and the TPR domain strongly inhibited the recruitment of (+)repRNA to the membrane (Figure 5B, lanes 6–8 and 12–14 versus 1), while the Cyp domain only had a small inhibitory effect (Figure 5B, lanes 9–11). These data strongly support that Cpr7p can inhibit (+)repRNA recruitment, which is critical for TBSV replication [29], [33], [44].

The second test focused on the assembly of the viral replicase, which can be performed in the CFE in the presence of ATP and GTP (Figure 5C). Then the assembled, membrane-bound replicase complex is solubilized with nonionic detergent, affinity-purified, and followed by testing its activity with added TBSV (−)repRNA [29], [31]. The data show strong inhibition of replicase activity by the full-length Cpr7p and the TPR domain (Figure 5D, lanes 2 and 4), suggesting that these proteins inhibited the *in vitro* assembly of the TBSV replicase complex *in vitro*.

The third assay tested the effect of recombinant Cpr7p and its derivatives on the (+) and (−)RNA synthesis in the CFE (Figure 5E). This assay is based on the previous observation that the ^32^P-labeled (−)RNA runs as dsRNA, while the newly made ^32^P-labeled (+)RNA migrates as single-stranded product in nondenaturing PAGE [29]. The *in vitro* results demonstrated that both the (+) and the (−) RNA synthesis was strongly inhibited by the full-length Cpr7p and the TPR domain, but the ratio of these RNAs did not change (Figure 5E). We interpret these data that RNA synthesis was inhibited by Cpr7p, but this could be either direct inhibition or the consequence of inhibition of the replicase assembly step (see above), which precedes RNA synthesis.

**Figure 6:**
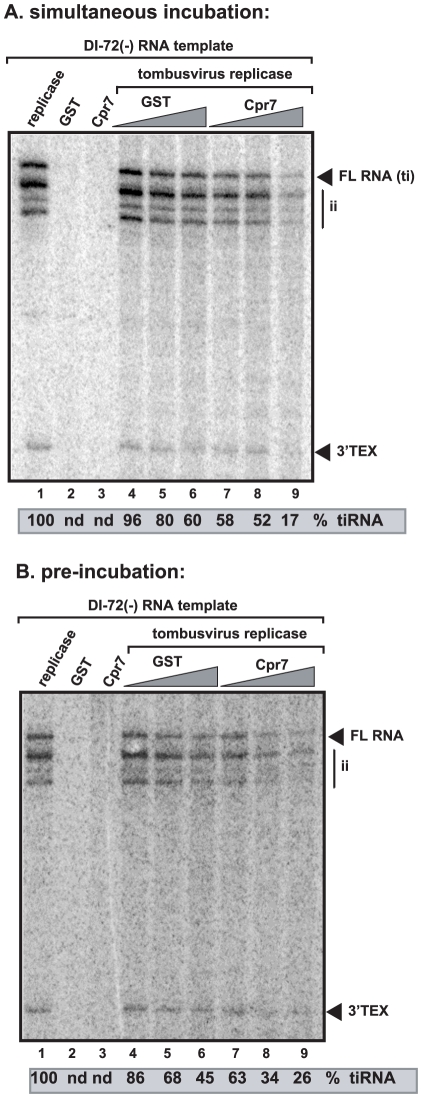
Cpr7p inhibits complementary RNA synthesis by the affinity-purified tombusvirus replicase. (A) Representative denaturing gel of ^32^P-labeled RNA products synthesized by the purified tombusvirus replicase *in vitro* in the presence of 0.8, 1.2 and 1.6 µg of purified recombinant Cpr7p or GST. The *in vitro* assays were programmed with DI-72 (−)repRNA, and they also contained ATP/CTP/GTP and ^32^P-UTP. All the components were added at the same time. The level of complementary RNA synthesis producing “FL” (the full-length product, made via initiation from the 3′-terminal promoter, also called “ti” product) is shown as % of FL product in the control sample. Note that this replicase preparation also synthesizes internal initiation products (“ii”) and 3′-terminal extension products (“3′TEX”). Each experiment was repeated three times. (B) The *in vitro* replicase experiment is similar to that in Panel A, except GST-Cpr7 or GST was pre-incubated for 10 min with the replicase to facilitate binding of Cpr7p and the VRC prior to RNA synthesis.

For the fourth assay, we utilized detergent-solubilized and affinity-purified tombusvirus replicase from yeast (Figure 6). This purified replicase lacks endogenous RNA, which is removed during purification, and can only synthesize complementary RNA products on added TBSV templates [20], [25], [36]. However, unlike the above membrane-bound replicase in the CFE-based assay, it cannot perform a complete cycle of RNA synthesis [20], [36]. The addition of purified recombinant Cpr7p to the purified tombusvirus replicase programmed with the (−)repRNA inhibited (+)-strand synthesis by up to ∼3-fold (Figure 6A, lanes 7–9 versus 4–6). Interestingly, Cpr7p inhibited not only the production of the full-length (+)-strand RNA product (produced via *de novo* initiation), but the amount of 3′-terminal extension product (3′TEX; due to self-priming by the 3′ end of the template [45], [46], [47]) as well. Therefore, we suggest that Cpr7p can inhibit both *de novo* initiation and 3′TEX on the (−)RNA template by the tombusvirus replicase.

Pre-incubation of Cpr7p with the purified replicase preparation, prior to the addition of the template (−)repRNA and the ribonucleotides to start complementary RNA synthesis, resulted in inhibition of RNA synthesis by ∼2-fold (Figure 6B, lanes 7–9 versus 4–6). These data demonstrate that Cpr7p likely associates rapidly with the tombusvirus replication proteins and pre-incubation is not needed to increase the strong inhibitory function of Cpr7p on tombusvirus replicase activity.

### Cpr7p binds to the RNA-binding region of the p33 replication protein

**Figure 7:**
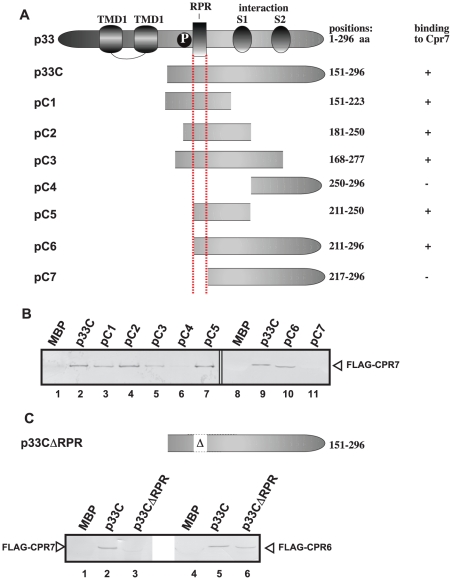
Binding of Cpr7p to TBSV p33 protein derivatives *in vitro*. (A) Schematic representation of viral p33 and its derivatives used in the binding assay. The various domains include: TMD, transmembrane domain; RPR, arginine-proline-rich RNA binding domain; P; phosphorylated serine and threonine; S1 and S2 subdomains involved in p33:p33/p92 interaction. (B) Affinity binding (pull-down) assay to detect interaction between FLAG-Cpr7p and the MBP-tagged viral p33 protein derivatives. The MBP-tagged viral proteins and MBP produced in *E. coli* were immobilized on amylose-affinity columns. Then, FLAG-Cpr7p expressed in BY4741 yeast was passed through the amylose-affinity columns with immobilized MBP-tagged proteins. The affinity-bound proteins were eluted with maltose from the columns. The eluted proteins were analyzed by Western blotting with anti-FLAG antibody to detect the amount of FLAG-Cpr7p specifically bound to MBP-tagged viral proteins. (C) Pull-down assay to detect interaction between FLAG-Cpr7p, FLAG-Cpr6p and the MBP-tagged p33CΔRPR, a mutated viral p33 protein lacking the RPR motif that binds to the viral RNA. See further details in Panel B.

To obtain information on how Cpr7p could inhibit RNA recruitment and replicase assembly as well as block the replicase function, we decided to map the Cpr7p binding site in the overlapping TBSV replication proteins. To this end, we have used pull-down experiments with immobilized MBP-p33 and its truncation derivatives (Figure 7A) from yeast extract containing FLAG-tagged Cpr7p. These experiments revealed that Cpr7p binds to the arginine-proline-rich (RPR) motif in p33 (compare pC4 and pC6 versus pC7, lanes 6,10–11, Figure 7B). Indeed, deletion of the RPR motif inhibited p33 binding to Cpr7p (p33CΔRPR, Figure 7C, lane 3). The RPR-motif is the well-characterized RNA-binding site in p33 and p92 replication proteins required for specific viral RNA recruitment and replicase assembly [33], [35], [44].

In contrast, Cpr6p, another Cyp40-like protein with TPR domains, which also binds p33 replication proteins (Figure 1A), does not bind to the RPR-motif in p33 (Figure 7C, lane 6), but instead to a region proximal to the C-terminus (Figure S2B–C). Based on this difference between Cpr6p and Cpr7p in binding to p33 (and likely the overlapping p92), we propose that Cpr7p-binding to the RPR-motif in p33 is required for the inhibitory effect of this cyclophilin on tombusvirus replication.

### Cpr7p also inhibits alfanodavirus replication in yeast

**Figure 8:**
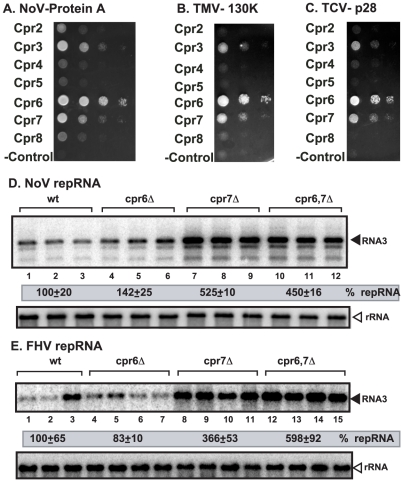
Increased NoV and FHV RNA accumulation in *cpr7*Δ yeast. Split ubiquitin MYTH assay was used to test binding between the yeast cyclophilins and NoV protein A (panel A), TMV 130K (panel B) and TCV p28 (panel C) replication proteins. The bait proteins were co-expressed with the prey cyclophilin proteins in yeast. The empty prey vector (NubG) was used as a negative control. To launch NoV (panel D) or FHV RNA1 (panel E) replication, we expressed NoV RNA1 from the copper-inducible *CUP1* promoter and FHV RNA1 and DI634 from the *CUP1* promoter in the parental (BY4741) and in *cpr6*Δ, and *cpr7*Δ single deletion and *cpr6*Δ *cpr7*Δ double deletion yeast strains. The yeast cells were cultured for 48 hours at 29°C in 3 ml SC-H^−^ with 2% glucose media containing 50 mM CuSO4 (for NoV) and for 24 hours at 29°C in 3 ml SC-H^−^ with 2% galactose (for FHV). Northern blot analysis was used to detect RNA1/RNA3 accumulation for NoV and FHV. The accumulation level of NoV and FHV RNAs was normalized based on 18S rRNA. Each experiment was repeated.

To test if replication of other RNA viruses could also be regulated by Cyp40 proteins, first we have performed MYTH assay with cyclophilins and replication proteins of plant and insect viruses. These experiments revealed that both Cpr7p and Cpr6p could interact with protein A of Nodamuravirus (NoV), an insect virus, p130 replication protein of *Tobacco mosaic virus* (TMV) and p28 of *Turnip crinkle virus* (TCV) (Figure 8A). In addition, we found that Cpr1p (Figure S4A–D) and Cpr3p (Figure 8A) can also interact with NoV protein A, TMV p130 and TCV p28, although the importance of these interactions was not studied further. Then, we tested if deletion of *CPR7* and *CPR6* could affect NoV and the related Flock house virus (FHV) replication in yeast model host [48]. Interestingly, NoV RNA replicated ∼5-fold more efficiently in *cpr7*Δ and *cpr6*Δ*cpr7*Δ yeast when compared with the wt yeast cells (Figure 8D, lanes 7–12 versus 1–3). Similar to TBSV, NoV RNA also replicated only slightly better in *cpr6*Δ yeast (Figure 8D, lanes 4–6) than the wt yeast, suggesting that *CPR6* is not as important as *CPR7* in regulation of NoV replication in yeast. We found similar pattern with FHV (Figure 8E), which is closely related to NoV, that *CPR7* deletion has a bigger effect than *CPR6* deletion on virus replication. Overall, these experiments confirmed that replication of two insect RNA viruses is also affected by Cpr7p, and that Cpr7p can interact with the replication protein of NoV in yeast. Therefore, it is possible that replication of other RNA viruses could be regulated by Cyp40 proteins.

## Discussion

Genome-wide screens and global proteomics approaches with tombusviruses unraveled many host proteins that can facilitate or inhibit virus infections [9], [11], [22]–[24], [29], [39], [49], [50]. One group of these host factors is cyclophilins, a large family of peptidyl-prolyl *cis-trans* isomerases with protein chaperone-like function that play a global role in facilitating correct protein folding and conformational changes in client proteins [40], [41]. Isomerization of peptidyl-prolyl bonds is frequently required for protein refolding, maturation and trafficking. Cyclophilins share a common 109 aa cyclophilin-like domain (CLD) performing the PPIase activity and additional domains unique to each member of the family. The unique domains, such as the TPR domain, are important for selection of protein substrates and subcellular compartmentalization. *S. cerevisiae* and *Arabidopsis* have 8 and 29 cyclophilins, respectively, while humans have 16 cyclophilin isoforms that have different cellular and tissue distribution [41], [43], [51].

We have found that four yeast cyclophilins (Cpr1p, Cpr3p, Cpr6p and Cpr7p), in addition to Fpr1p (Figure 1) and Ess1p PPIases [39], interacted with p33. CFEs prepared from a yeast strain lacking these genes (except *ESS1*, which is essential for yeast growth) supported TBSV replication *in vitro* at a higher level than CFE from wt yeast, or a yeast strain lacking the remaining noninteracting members of cyclophilin and FKB genes. Altogether, the data firmly established that the group of cyclophilins that interacts with p33 is a robust negative regulator of TBSV replication. Among the cyclophilins, we have discovered that the yeast Cyp40-like Cpr7p strongly inhibits TBSV replication in yeast model host and *in vitro* in a TBSV replication assay based on CFE. Three Cyp40-like proteins from *Arabidopsis* plant also interacted with p33/p92 replication proteins and they showed remarkable inhibitory effect on TBSV repRNA accumulation when added to the CFE-based replication assay (Figure 3C and Figure S3). Thus, the inhibitory effect of Cyp40-like proteins against TBSV is conserved between *S. cerevisiae* and *Arabidopsis*. Also, it seems that Cpr7p can also inhibit the replication of insect RNA viruses NoV and FHV (Figure 8) [52], which are distantly related to TBSV. Based on these data, we propose that Cyp40-like proteins could be important inhibitors or regulators of various RNA viruses. Interestingly, Cyp40 is required for the activity of microRNAs in plants [53]. Thus, in addition to the regulatory role in RNA viruses, Cyp40 is also involved in other RNA-based processes.

**Figure 9:**
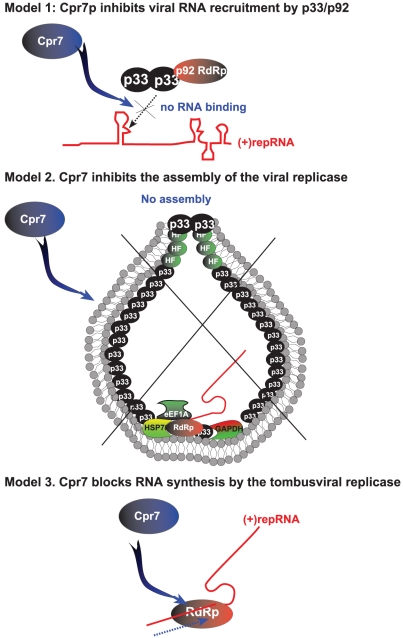
Models for the role Cpr7p in tombusvirus replication. Model 1: Cpr7p has been shown to inhibit the recruitment of the viral RNA into replication. Model 2: The assembly of the tombusviral VRC requires interactions among the viral replication proteins, selected host proteins, such as Hsp70, GAPDH or eEF1A or membrane lipids. Cpr7p might inhibit any of these interactions, resulting in reduced TBSV RNA replication. In addition, Cpr7p binds to the RPR RNA binding motif in p33 and likely inhibiting p33: viral (+)RNA interaction that is needed for VRC assembly. Model 3: Due to the chaperone activity of the TPR domain, binding of Cpr7p to p92 might inhibit the RdRp function of p92, thus blocking RNA synthesis. See further details in the text.

The inhibitory effect of Cpr7p on TBSV replication seems to depend on the TPR-domain, which can specifically bind to the RNA-binding region of p33/p92 replication proteins of TBSV (Figure 7). Indeed, Cpr7p or the TPR domain: p33 interaction inhibits the recruitment of the viral RNA to membranes (Figure 9), where viral replication takes place. In addition, Cpr7p also inhibits the assembly of the functional VRC *in vitro* (Figure 9), which then leads to decreased level of viral RNA synthesis [both (+) and (−)RNA synthesis is inhibited proportionately, Figure 5E]. Inhibition of VRC assembly could be due to binding of Cpr7p to the RPR RNA binding motif, thus inhibiting the RNA binding activity of p33 and p92. Without the bound (+)RNA, p33 and p92 cannot assemble functional VRC since multiple *cis*-acting elements within the TBSV (+)RNA are essential for VRC assembly [36], [54]. However, we cannot exclude that Cpr7p might inhibit VRC assembly indirectly via inhibition of viral (+)RNA recruitment, which is required for VRC assembly [20], [36]. In addition, recombinant Cpr7p binding to the purified tombusvirus replicase also results in reduced RNA synthesis *in vitro*, indicating that Cpr7p can block viral RNA replication at several steps (Figure 9). Interestingly, Cpr7p cannot efficiently inhibit the pre-assembled, membrane-bound TBSV replicase in a CFE-based assay (Figure 4C), suggesting that Cpr7p has to be present at the early stage of replicase assembly for robust inhibition. It is likely that the membrane-bound VRC might not be accessible for Cpr7p after the VRC assembly is completed in CFE.

Similar to Cpr7p, another Cyp40-like protein (i. e., Cpr6p with 38% sequence identity with Cpr7p) can also bind to p33/p92 replication proteins (Figure 1 and S2B). However, this binding does not lead to significant inhibition of TBSV replication in yeast (Figure 2) or in the CFE-based TBSV replication assay (Figure S1C). Since the Cpr6p : p33 interaction does not involve the RNA-binding region in p33 (Figure S2B), we propose that this interaction is less inhibitory to TBSV replication by likely allowing p33 to perform its important functions in viral RNA recruitment and VRC assembly as well as its RNA chaperone activity [33], [55]. Another possibility for the weak inhibitory effect of Cpr6p is its low chaperone activity. Accordingly, it has been shown that Cpr6p and Cpr7p have different activities: Cpr6p is far more active as a PPIase, while Cpr7p is a much better chaperone than Cpr6p [56]. Indeed, *CPR6* could not complement the growth defect caused by deletion of *CPR7* in yeast and the defined functions of Cpr7p in signaling and HSP90 binding are dependent on the TPR domain [56]–[58]. Interestingly, Cpr6p can also interact with protein A of NoV without effective inhibition of NoV replication (Figure 8). Thus, it seems that Cpr6p has evolved different functions from Cpr7p, including the regulatory role in viral RNA replication. In contrast, all three *Arabidopsis* Cyp40-like proteins are inhibitory to TBSV replication *in vitro* (Figure 3), suggesting that plants might have more cyclophilins to combat some viral infections than yeast does.

Similar to our findings with Cyp40 homologs, other cyclophilins have been shown to inhibit accumulation of several RNA viruses. For example, CypA was found to bind to the matrix protein (M1) of influenza A virus and interfered with the nuclear localization of M1 [59]. Over-expression of CypA increased the self-association of M1 protein and led to decreased viral replication. Similarly, CypA was also found to inhibit the infectivity of HIV-1 (human immunodeficiency virus-1) virions [60], [61]. Interestingly, due to retrotransposition of *CypA* between exon 7 and 8 in *TRIM5* gene in New World owl monkeys, a CypA-TRIM5 fusion protein has emerged, which targets the HIV-1 coat protein causing resistance to HIV-1 infections [62], [63]. CypA gets incorporated into HIV-1 virions via direct binding to Gag, the polyprotein precursor of virion structural proteins [61], [64], [65]. The role of CypA as an anti-HIV protein is neutralized by the retroviral Vif protein, which inhibits the incorporation of CypA into the viral particles [66]. A genome-wide screen for host factors affecting West Nile virus identified FKBP1B immunophilin as an inhibitor of this virus [67]. Overall, cyclophilins/immunophilins are potent inhibitors of several RNA viruses and they might be part of the innate response of the host against some viruses. Since PPIases are conserved and ubiquitous proteins, their role could be common against viruses.

On the contrary, several RNA viruses seem to hijack cyclophilins to facilitate their replication. For example, cyclosporine, an inhibitory peptide of cyclophilins, markedly inhibited HCV replication in cell cultures [68], [69]. It has been proposed that the cytosolic CypA affects the assembly of the HCV VRC by likely influencing the cleavage of NS5A-NS5B fusion protein and the folding of the NS5A and NS5B RdRp proteins [70]–[72]. CypA seems to affect multiplication of several flaviviruses, such as WNV, Dengue virus and yellow fever virus [73]. CypA was shown to bind to the NS5 replication protein and is likely component of the VRC and cyclosporine has been shown to inhibit flaviviral RNA synthesis [73].

The human FKBP8 immunophilin, which interacts via the three TPR repeats with the HCV NS5A is also important for HCV replication [74]. FKBP8 might function in HCV replication by forming a complex between NS5A, FKBP8 and Hsp90 [74]. Interaction between NS5A and FKBPs is also important for HCV to block apoptosis in hepatoma cells [75].

Overall, cyclophilins and other PPIases seem to play important roles during RNA virus infections. The current work revealed a new role for Cyp40-like proteins and their TPR domains as inhibitors and regulators of RNA virus replication. This function for Cyp40 seems to be conserved between yeast and plants and also effective against tombus- and alfanodaviruses.

## Materials and Methods

### Yeast strains and expression plasmids

The following yeast strains with deletion of various cyclophilin genes were used in this study: the wild type #149 (JK93da); 163: KDY81.18C (*fpr1-4*Δ);164: KDY97.19 (*cpr1-8*Δ); 165: SMY101-1 (*cpr2,4,5,8*Δ*/fpr2*Δ); 166: KDY98.49 (*cpr1-8*Δ*/fpr1-4*Δ) (generous gift of Joseph Heitman) [76]. Yeast strain 168 (*cpr1,3,6,7*Δ*/fpr1*Δ) was created by homologous recombination based on strain KDY75.3b using the HYG gene to replace *CPR3*. PCR was performed using plasmid pFA6-hphNT1 (EUROSCARF) [77] as template and primer set #3279/#3280 (Table S1). The PCR product was transformed to KDY75.3b yeast strain and recombinant yeast colonies were selected in SC-LA^−^ plates supplemented with G418 and hygromycin.

The plasmids pHisGBK-CUP-His33/GAL-DI72 and pGAD-CUP-Hisp92 used for viral replication experiments in yeast, have been previously described [26], [78]. Plasmids pGAD-ADH-HFp92, pYC-GAL-DI72, and pHisGBK-ADH-HFp33 were generated by Serva and Nagy [25].

To generate the *Escherichia coli* expression plasmids for GST-CPR7 and its truncated derivatives, CPR7-CYP and CPR7-TPR, we PCR-amplified the full-length sequence of *CPR7* as well as *CPR7-CYP* and *CPR7-TPR* domains (Figure 3B) with primer sets #3152/#4116, #3152/#4132, and #4131/#4116, respectively. To generate the *E. coli* expression plasmids for GST-At5g48570 and GST-At5g48570-TPR, we PCR amplified the full-length sequence of At5g48570 and At5g48570-TPR with primer sets #4332/#4333 and #4374/#4375 (Table S1), respectively. The PCR products were digested with *BamH*I and *EcoR*I and ligated into pGEX-2T using the same enzymes.

The FHV expression plasmid pESC-His/gal/FHV/RNA1 has been generated before [48]. To launch NoV RNA1 replication in yeast, we generated plasmid pEsc-His/Cupm/NoV/RNA1/TRSVrz that expressed the full-length NoV RNA1 via cleavage of the transcribed RNA1 at the 3′ end by a ribozyme, similar to the strategy used for FHV RNA1 expression [48]. Three PCR products were ligated to each other in a stepwise fashion. The first fragment was amplified from pGAD/CupHis92 with primers #3873 and #3861 to obtain a modified *CUP1* promoter with a predicted transcriptional start site at or around the first viral nucleotide. The second fragment was amplified from pMT/NoVRNA1 (provided by SW Ding) with primers #3862 and #3863 to obtain the full-length cDNA for NoV RNA1. The third fragment was generated with primers #3871 and #3875 from pYC/DI72/sat to amplify the ribozyme. The PCR reactions were carried out with Phusion polymerase. Primers #3861, #3862, #3863 and #3871 were kinased with T4 polynucleotide kinase prior to PCR. The final PCR was carried out with primers #3873 and #3875 using High Fidelity Taq polymerase (Invitrogen). The resulting PCR product was digested with BglII and NheI and cloned into pEsc-His (Agilent) digested with BamHI and BcuI.

### Analysis of protein-protein interaction using the split-ubiquitin assay

The split-ubiquitin assay is based on the Dualmembrane kit3 (Dualsystems) and performed as previously described [39], [79]. The bait constructs, pGAD-BT2-N-His33 and pGAD-BT2-N-His92, expressing tombusvirus replication proteins p33 and p92, respectively, were as described [26], [39]. The *CPR7* and the truncated PCR products of *CPR7* were digested with *Bam*HI and *Nhe*I and ligated into the pPRN-N-RE (Dualsystems) vector digested with the same enzymes to generate pPRN-N-CPR7. Yeast strain NMY51 was co-transformed with pGAD-BT2-N-His33 and pPR-N-RE (NubG, as a negative control) or one of the prey-constructs (pPR-N-CPR7) and plated onto Trp−/Leu− (TL^−^) synthetic minimal medium plates for plasmid selection. Yeast colonies were then re-suspended in 50 µl water and spotted onto Trp−/Leu−/His−/Ade− (TLHA^−^) plates to detect p33 : Cpr7p interactions as described [26]. Plasmid expressing Ssa1p protein (pPR-N-SSA1) was used as a positive control as previously described [39], [79].

### Analysis of protein-protein interaction *in vitro*

The MBP-tagged p33 and p33C were purified from *E. coli* as described previously [80]. *E. coli* cells were resuspend in cold column buffer (10 mM Tris-HCl [pH 7.4], 1 mM EDTA, 25 mM NaCl, 10 mM β-mercaptoethanol) and broken by sonication. The cleared lysate was passed through an amylose column to bind the MBP-tagged viral proteins or MBP (negative control). The columns were washed three times with cold column buffer prior to addition of the yeast lysate. For the pull-down assay, 100 mg of yeast pellets containing FLAG-tagged Cpr7p (pESC-His-CupFlag-CPR7) or its truncated forms were resuspended in 150 µl pre-chilled Buffer I (20 mM Tris-HCl [pH 7.5], 1 mM EDTA, 200 mM NaCl, 10% [V/V] glycerol, 0.1% [V/V] NP40, 10 mM β-mercaptoethanol, 1% [V/V] yeast protease inhibitor cocktail (Ypic)]) and 1 µl of RNase A (1 mg/ml). Yeast cells were broken in Genogrinder with 250 µl volume of acid washed glass beads for 2 min at 1,500 rpm followed by addition of 600 µl of Buffer I. The yeast lysate was centrifuged at 100× g at 4°C for 5 min, and the supernatant was transferred to a pre-chilled eppendorf tube and centrifuged at 15,000 rpm at 4°C for 5 min before loading onto the amylose columns and incubated for 3 h at 4°C. The columns were washed five times with cold column buffer II (20 mM Tris-HCl [pH 7.5], 1 mM EDTA, 100 mM NaCl, 10% [V/V] glycerol, 0.1% [V/V] NP40), and the bound protein complexes were eluted with column buffer supplemented with 10 mM maltose. The presence of FLAG-Cpr7p protein in the eluate was analyzed by sodium dodecyl sulfate-polyacrylamide gel electrophoresis (SDS-PAGE) followed by Western blotting using an anti-Flag antibody (Sigma). The amount of MBP-tagged viral proteins in the eluate was visualized by Coomassie blue staining of the 12% SDS-PAGE gels.

### Analysis of TBSV, NoV and FHV RNA replication in yeast

For measuring TBSV repRNA accumulation, yeast strain BY4741, *cpr6*Δ, *cpr7*Δ, and *cpr6,7*Δ were transformed with plasmids pGAD-CUP-Hisp92, pHisGBK-CUP-Hisp33/GAL-DI72. Replication assay was performed by measuring the accumulation of DI-72(+) repRNA (normalized to the 18S rRNA). Replication was induced by adding 50 µM CuSO_4_ to the SC-LH^−^ medium and then yeast was cultivated for 24 h at 23°C or 29°C. For measuring NoV RNA1/3 accumulation, yeast strain BY4741, *cpr6*Δ, *cpr7*Δ, and *cpr6,7*Δ were transformed with plasmid pESC-His/Cupm/NoV/RNA1/TRSVrz. Replication was induced by adding 50 µM CuSO_4_ to the SC-H^−^ medium containing 2% glucose and then yeast was cultured for 48 h at 29°C. For measuring FHV RNA1/3 accumulation, yeast strain BY4741, *cpr6*Δ, *cpr7*Δ, and *cpr6,7*Δ were transformed with plasmids pESC-URA/Cupm/FHV/DI634/TRSVrz and pESC-His/Gal/FHV/RNA1/TRSVrz. Replication was induced by adding 50 µM CuSO_4_ to the SC-UH^−^ medium containing 2% galactose and then yeast was cultured for 24 h at 29°C.

### Yeast CFE-based TBSV replication assay

The different yeast strains used for preparation of CFEs were grown in YPD medium at 29°C for 6–8 h to a final OD 1.5 followed by 30 min at 37°C before harvest and prepared as described [31]. A total reaction volume (25 µl) contained 3 µl of whole cell extract, 0.5 µg DI-72 (+)repRNA transcript, 700 ng purified MBP-p33, 1.4 µg purified MBP-p92^pol^ (both recombinant proteins were purified from *E. coli*, see below), 30 mM HEPES-KOH, pH 7.4, 150 mM potassium acetate, 5 mM magnesium acetate, 0.13 M sorbitol, 0.4 µl actinomycin D (5 mg/ml), 2 µl of 150 mM creatine phosphate, 0.2 µl of 10 mg/ml creatine kinase, 0.2 µl of RNase inhibitor, 0.2 µl of 1 M dithiothreitol (DTT), 2 µl of 10 mM ATP, CTP, and GTP and 0.25 mM UTP and 0.1 µl of [^32^P]UTP [35]. The reaction mixture was incubated at 25°C for 3 h and terminated by adding 100 µl stop buffer (1% sodium dodecyl sulfate [SDS] and 0.05 M EDTA, pH 8.0) followed by phenol-chloroform extraction, isopropanol-ammonium acetate precipitation overnight at −20°C and a washing step with 70% ethanol as described [81]. The newly synthesized ^32^P-labeled RNA products were separated by electrophoresis in a 5% polyacrylamide gel (PAGE) containing 0.5× Tris-borate-EDTA (TBE) buffer with 8 M urea. Signals were detected using a Typhoon 9400 imaging scanner (Amersham) and quantified by imageQuant software.

### Testing the effect of recombinant Cpr7 in yeast CFE-based TBSV replication assay

Reaction mixtures for *in vitro* replicase assays were set-up according to the *in vitro* replication assay previously described [31], [35] with the following modification. In addition to the recombinant viral replication proteins, MBP-p33 and MBP-p92^pol^, 800 ng of GST-Cpr7p or its deletion derivatives were also included in the assay. Reactions containing only GST and CFE served as negative control. The assays included increasing amounts (0.4, 0.8 and 1.6 µg) of purified recombinant proteins, such as GST-Cpr7p (5.6, 11.3 and 22.5 µM), GST-CYP (7.6, 15.3 and 30.5 µM), GST-TPR (8.3, 16.7 and 33.4 µM) or GST (15.4, 30.7 and 61.5 µM).

### Protein purification from *E. coli*

Expression and purification of the recombinant TBSV p33 and p92 replication proteins from *E. coli* were carried out as described earlier with modifications [80], [82]. The expression plasmids were transformed separately into *E. coli* strain BL21DE3CodonPlus. Protein expression was induced using isopropyl β-D-thiogalactopyranoside (IPTG) for 8 h at 16°C, and the cells were harvested by centrifugation at 5,000 rpm at 4°C for 5 min to remove the medium prior to −80°C storage. Affinity columns containing amylose resin (NEB) were used to purify MBP-tagged recombinant proteins as described [80], [82]. Purification of GST-tagged proteins (pGEX-) was carried out using glutathione resin and eluted with 10 mM glutathione, 10 mM ß-mercaptoethanol in the column buffer following the same protocol as MBP-proteins. Briefly, the frozen pellets were suspended and sonicated in MBP column buffer containing 20 mM Tris-Cl pH 8.0, 150 mM NaCl, 1 mM EDTA, 10 mM β-mercaptoethanol and 1 mM phenylmethylsulfonyl fluoride (PMSF). The sonicated extract was centrifuged at 15,000 rpm for 5 min, and the supernatant was added to the pre-equilibrated amylose resin for 1 h rotating incubation at 4°C. After washing the resin 3 times with column buffer and once with a low salt column buffer (25 mM NaCl), the proteins were eluted with a low salt column buffer containing 0.18% (V/W) maltose and 6% (V/V) glycerol and aliquoted for storage at −80°C. The concentration of the purified recombinant proteins was measured by Bio-Rad protein assay. Protein fractions used for the replication assays were 95% pure, as determined by SDS-PAGE.

### *In vitro* tombusvirus replicase assay with affinity purified replicase preparations

Yeast BY4741 strain was transformed with the plasmids pGAD-ADH-HFp92, pYC-GAL-DI72, and pHisGBK-ADH-HFp33 expressing 6xHis- and Flag-tagged tombusvirus p33 and p92. Initially, yeast transformants were grown in 10 ml of ULH^−^ selective medium overnight at 30°C then transferred to fresh 200 ml ULH^−^ medium to 0.2 OD_600_ and grown at 23°C until 1.5 OD_600_. FLAG-affinity-purification was done according to a previously described procedure with the following modification [24]. Briefly, 2 g of yeast cells were resuspended and homogenized in TG buffer [50 mM Tris–HCl [pH 7.5], 10% glycerol, 15 mM MgCl_2_, 10 mM KCl, 0.5 M NaCl, 0.5% Triton, and 1% [V/V] yeast protease inhibitor cocktail (Ypic)] by glass beads using FastPrep Homogenizer (MP Biomedicals). The yeast cell lysate was cleared by centrifugation at 500× g for 5 min at 4°C to remove unbroken cells and debris. The membrane fraction containing the viral replicase complex was collected by centrifugation at 35,000× g for 15 min at 4°C and then solubilized in 1 ml TG buffer with a buffer containing 2% Triton, 1% [V/V] Ypic via gentle rotation for 3 h at 4°C. The solubilized membrane fraction was centrifuged at 35,000× g for 15 min at 4°C and the supernatant was incubated with 100 µl anti-FLAG M2-agarose affinity resin (Sigma) pre-equilibrated with 1 ml TG buffer. After 3 h of gentle rotation at 4°C, the column was washed 5 times with TG buffer containing 0.5% Triton. The resin-bound replicase complex was eluted in 700 µl elution buffer [50 mM Tris–HCl [pH 7.5], 10% glycerol, 15 mM MgCl_2_, 10 mM KCl, 0.05 M NaCl, 0.5% Triton, 1% Ypic and 0.15 mg/ml FLAG peptide (Sigma)] following overnight rotation at 4°C. *In vitro* replicase activity of the purified preparations was tested using DI-72(−) RNA template transcribed *in vitro* by T7 transcription [24].

### *In vitro* viral RNA recruitment assay

The *in vitro* RNA recruitment assay based on yeast CFE was performed according to [31], [35], except that ^32^P-labeled DI72 (+) repRNA were used and rCTP, rUTP, ^32^P-labeled UTP, and Actinomycin D were omitted from the assay. As a negative control, GST protein purified from *E. coli* was used in yeast CFE in the absence of p33/p92. Following two-hour incubation at 25°C, 1 ml of reaction buffer was added to the *in vitro* reaction mixture and further incubated on ice for 10 min before centrifugation at 35,000× g for 45 min. The membrane-bound ^32^P-labeled (+)repRNA was extracted from the pellet by adding 0.1 ml stop buffer and 0.1 ml phenol/chloroform followed by brief vortex at high setting and centrifuged at 27,000× g for 4 min. The supernatants from each reaction were precipitated with isopropanol/ammonium acetate overnight at −20°C. The RNA samples were analyzed by denaturing PAGE and phosphoimaging as described [31], [35].

### Northern and Western blot analyses

Total-RNA isolation and Northern blot analysis were performed as described previously [21]. The probes for Northern blot to detect FHV RNA1/RNA3 (annealing to the 3′ end of FHV RNA1) and NoV RNA1/RNA3 (annealing to the 3′ end of NoV RNA1) was obtained via T7 transcription on PCR templates generated by using the following primers: #3676 and #3675 (for FHV) and #3867 and #3868 (for NoV) (Table S1).

Protein analysis was performed as described previously using an anti-His_6_ antibody [20] as the primary antibody for the detection of His_6_-p33 and His_6_-p92. Detection of FLAG-CPR7 and its truncated derivatives were carried out using primary anti-FLAG antibody following the manufacturer's instructions (Sigma). The secondary antibody for both primary antibodies was alkaline phosphatase-conjugated anti-mouse immunoglobulin G (Sigma).

Additional Materials and Method section is available as Text S1.

## Supporting Information

Figure S1 CFE-based TBSV replication assay demonstrates an inhibitory role for Cpr3p and Cpr7p. (A) Top panel: Denaturing PAGE analysis of the ^32^P-labeled TBSV repRNA products obtained in the CFE-based assay programmed with in vitro transcribed TBSV DI-72 (+)repRNA and purified recombinant p33 and p92^pol^ replication proteins of TBSV. Purified recombinant MBP-tagged Cpr1p, Cpr3p, Cpr7p, and Fpr1p (0.1, 0.2 and 0.4 µg), or MBP were added to CFE prepared from BY4741 yeast strain. Each experiment was repeated three times. Bottom panel: Ethidium-bromide stained PAGE gel from the top panel to show the sample loading and the lack of RNase activity in the CFE-based assay. (B) SDS-PAGE analysis of the purified recombinant proteins used in the above CFE-based assay. (C) Denaturing PAGE analysis of the ^32^P-labeled TBSV repRNA products obtained in the CFE-based assay programmed with in vitro transcribed TBSV DI-72 (+)repRNA and purified recombinant p33 and p92^pol^ replication proteins of TBSV. Two-step purified recombinant 6xHis-GST-tagged Cpr6p and Cpr7p (0.4, 0.8 and 1.6 µg), or GST were added to CFE prepared from BY4741 yeast strain. Each experiment was repeated three times. See additional details in Panel A. (D) SDS-PAGE analysis of the two-step purified recombinant 6xHis-GST-tagged Cpr6p and Cpr7p proteins used in the above CFE-based assay.
(EPS)

Figure S2 Binding of Cpr6p to TBSV p33 protein derivatives. (A) Split ubiquitin assay was used to test binding between p33 and the full-length Cpr6p and its two domains (Cyp and TPR, respectively). The bait p33 was co-expressed with the prey proteins in yeast. Ssa1p (HSP70 chaperone), and the empty prey vector (NubG, shown as “-control”) were used as positive and negative controls, respectively. (B) Schematic representation of viral p33 and its derivatives used in the binding-assay. The various domains include: TMD, transmembrane domain; RPR, arginine-proline-rich RNA binding domain; P; phosphorylated serine and threonine; S1 and S2 subdomains involved in p33:p33/p92 interaction. (C) Affinity binding (pull-down) assay to detect interaction between FLAG-Cpr6p and the MBP-tagged viral p33 protein derivatives. The MBP-tagged viral proteins and MBP produced in *E. coli* were immobilized on amylose-affinity columns. Then, FLAG-Cpr6p expressed in BY4741 yeast was passed through the amylose-affinity columns with immobilized MBP-tagged proteins. The affinity-bound proteins were eluted with maltose from the columns. The eluted proteins were analyzed by Western blotting with anti-FLAG antibody to detect the amount of FLAG-Cpr6p specifically bound to MBP-tagged viral proteins. (D) Detection of *CPR7* mRNA levels in various yeast strains by semi-quantitative RT-PCR. The top panel shows an ethidium-bromide-stained 1.5% agarose gel of the semi-quantitative RT-PCR products. The bottom panel shows an ethidium-bromide-stained 1.5% agarose gel of the total RNAs as loading controls.
(EPS)

Figure S3 CFE-based TBSV replication assay demonstrates an inhibitory role for the *Arabidopsis* Cyp40-like proteins. (A) Denaturing PAGE analysis of the ^32^P-labeled TBSV repRNA products obtained in the CFE-based assay programmed with in vitro transcribed TBSV DI-72 (+)repRNA and purified recombinant p33 and p92^pol^ replication proteins of TBSV. Purified recombinant GST-tagged full-length Cyp40s or their TPR domains (0.4, 0.8 and 1.6 µg), were added to CFE prepared from BY4741 yeast strain. Each experiment was repeated three times. (B–C) Split ubiquitin MYTH assay was used to test binding between the TPR domain of three *Arabidopsis* Cyp40 proteins and the TBSV p33 (panel B) and p92 (panel C) replication proteins. The bait proteins were co-expressed with the prey cyclophilin proteins in yeast. The empty prey vector (NubG) was used as a negative control. Note the weak, but detectable interaction between At2g15790-TPR and p33/p92 proteins.
(EPS)

Figure S4 Split ubiquitin MYTH assay was used to test binding between the yeast Cpr1p and the following viral replication proteins: NoV protein A (panel A), TMV 130K (panel B), TCV p28 (panel C) and the p33 of *Cucumber necrosis virus* (CNV, a tombusvirus, closely related to TBSV) (panel D). The bait proteins were co-expressed with the prey Cpr1p in yeast. The other three host proteins were used as controls.
(EPS)

Table S1 The list of primers used in this study.
(DOC)

Text S1 Materials and methods.
(DOC)
