# Supplementary material for: The TPR Domain in the Host Cyp40-like Cyclophilin Binds to the Viral Replication Protein and Inhibits the Assembly of the Tombusviral Replicase
Source: PLoS Pathog. 2012 Feb 9;8(2):e1002491. doi: 10.1371/journal.ppat.1002491 (PMC3276564; doi:10.1371/journal.ppat.1002491)
Supplement: Table S1 — The list of primers used in this study. (DOC) [file ppat.1002491.s005.doc]

**Table S**1

| Primers | Sequence |
| --- | --- |

| 3142 | CGCGGATCCATGAAATTCAGTGGCTTGTGG |
| --- | --- |
| 3143 | CTAGCTAGCAGAAGAGAGCTCAGGCGTCCA |
| 3144 | CGCGGATCCATGTTTAAACGTTCCATCATT |
| 3145 | CTAGCTAGCTAACTCACCAGCTTCTTCGAT |
| 3146 | CGCGGATCCATGTGGTTGAAATCCTTGCTG |
| 3147 | CTAGCTAGCCTTGTGTCTTAATGAAACCGT |
| 3150 | CGCGGATCCATGACTAGACCTAAAACTTTT |
| 3151 | CTAGCTAGCGGAGAACATCTTCGAAAGAGA |
| 3152 | CGCGGATCCATGATTCAAGATCCCCTTGTA |
| 3153 | CTAGCTAGCGGAGAAAAACTTTGATATATT |
| 3154 | CGCGGATCCATGAAGAGCTTTTTTCTTTAT |
| 3155 | CTAGCTAGCATCCTCTCTAATAGAAACTAT |
| 3156 | CGCGGATCCATGTCTGAAGTAATTGAAGGT |
| 3157 | CTAGCTAGCGTTGACCTTCAACAATTCGAC |
| 3158 | CGCGGATCCATGATGTTTAATATTTACCTT |
| 3159 | CTAGCTAGCGGCGGCTGATTTCACGTCTAC |
| 3160 | CGCGGATCCATGTCTGATTTGTTACCACTA |
| 3161 | CTAGCTAGCGTTTTTCATAGAAACCAATTT |
| 3162 | CGCGGATCCATGTCTGATATGCTTCCATTG |
| 3163 | CTAGCTAGCTTTCATGGAGACCAATTTAAC |
| 3177 | CCGGAATTCATGAAGCTTCAATTTTTTTCC |
| 3178 | CCCATCGATGAGTTCATCGTGGGCTGCTTC |

| 3196 | ccgctcgagttaggagaaaaactttgatat |
| --- | --- |
| 3279 | TTATTTTAGTCCTTTCGCACCGCGATTATTAATAAACTCAATAcgtacgctgcaggtcga |
| 3280 | ACCTTATATGCTAGAAATATGAGAGACCTTAGCTTCATATAAatcgatgaattcgagctc |
| 3675 | gTAATACgACTCACTATAggagacctctgccctttcgggctagaac |
| 3676 | Catcactgctggagaaacccagcg |
| 3867 | cgggtccgccagctaaaacaacag |
| 3868 | gTAATACgACTCACTATAGaccactggcataagcctagttcg |
| 3861 | TATATGATATTGCACTAACAAGAAG |
| 3862 | gtattgaatccaaaactcaaaatgctg |
| 3863 | accactggcataagcctagttcg |
| 3871 | GTCCTGTTTCtTgCcAAACAGAGAAccacACCAGAGAAACACACGTTGTGG |
| 3873 | ccagAGATCTGACATTTGGGCGCTATACGTGCATATGT |
| 3875 | CCagGCTAGCctcTACCAGGTAATATACCACAACGTGTGT |
| 4116 | Cgccgaattcttaggagaaaaactttgatatatt |
| 4131 | Ggcgggatccgtgtgggaaaaaactatgggtgtcc |
| 4132 | Cgccgaattcttaagcagcctcaagagccttacc |
| 4332 | ggcgggatccatggaagacgatttcgacacg |
| 4333 | cgccgaattctcatgccttggtgtcaatact |
| 4374 | ggcgggatccatgaaagagtcttgggatatgaac |
| 4375 | cgccgaattcctagttgctgtaaaacttagcatc |
