# Supplementary material for: The TPR Domain in the Host Cyp40-like Cyclophilin Binds to the Viral Replication Protein and Inhibits the Assembly of the Tombusviral Replicase
Source: PLoS Pathog. 2012 Feb 9;8(2):e1002491. doi: 10.1371/journal.ppat.1002491 (PMC3276564; doi:10.1371/journal.ppat.1002491)
Supplement: Text S1 — Materials and methods. (DOC) [file ppat.1002491.s006.doc]

**Text S1. Materials and methods.**

**Yeast strains and expression plasmids.** To generate pET30a(+)/GST-CPR6 and pET30a(+)/GST-CPR7, the *Escherichia coli* expression plasmids for His-GST-CPR6 and His-GST-CPR7, the restriction sites used for ligation into pET30a(+) vector were EcoRV and XhoI. PCR amplification of GST-CPR6 and GST-CPR7 were performed with primers sets #4870/#3192 (5’-ccgctcgagtcaggagaacatcttcgaaag) and #4870 (5’-ggctgatatcatgtcccctatactaggttat**)**/#3196, respectively. PCR products were subcloned between the EcoRV and XhoI sites of pET30a(+).

**Protein purification from *E. coli.*** Expression plasmids pET30a(+)/GST-CPR6 and pET30a(+)/GST-CPR7 were introduced into a competent *E.coli* BL21DE3Codon Plus cells using standard protocols. Expression of His-GST-CPR6 and His-GST-CPR7 fusion protein was induced with 0.1 mM IPTG at 23°C for 6 h. The recombinant proteins were then purified using Ni Sepharose column. After washing the resin with different concentrations of imidazole washing buffer (20 mM phosphate, 500 mM NaCl and 20 mM/40 mM/60 mM/ 100 mM imidazole), the proteins were eluted with the elution buffer (20 mM phosphate, 500 mM NaCl, and 500 mM imidazole). Then, the eluted proteins were subjected to an additional purification on glutathione resin, followed by elution with 10 mM glutathione, 10 mM ß-mercaptoethanol in the column buffer following the same protocol as descried in the main text.

**Analysis of protein-protein interaction using the split-ubiquitin assay.** The full-length yeast cyclophilin/immunophilin genes were PCR-amplified using the following primer pairs: *CPR2*: #3142/3143; *CPR3*: #3144/3145; *CPR4*: #3146/3147; *CPR5*: #3177/3178; *CPR6*: #3150/3151; *CPR7*: #3152/3153; *CPR8*: #3154/3155; *FPR1*: #3156/3157; *FPR2*: #3158/3159; *FPR3*: #3160/3161; and *FPR4*: #3162/3163 (Table S1). The obtained PCR products were digested with *Bam*HI and *Nhe*I and ligated into the pPRN-N-RE (Dualsystems) vector digested with the same enzymes to generate pPRN-N-CPR2-8 or pPRN-N-FPR1-4. pPRN-N-CPR5 was generated by digesting the PCR product with *EcoR*I and *Cla*I and ligated into the pPRN-N-RE. pPRN-N-CPR1 was constructed before (ref. 39).

**Detection of  *CPR7* mRNA expression level.** Total RNAs were extracted from different yeast strains (BY4741, *cpr6*Δ, *cpr7*Δ, and *cpr6,7*Δ) and RT-PCR were performed using primers #3152/#4116 (Table S1). We performed 25 PCR cycles. The RT-PCR products were analyzed by 1.5% agarose gel.
